# Imaging of Red-Shifted Light From Bioluminescent Tumors Using Fluorescence by Unbound Excitation From Luminescence

**DOI:** 10.3389/fbioe.2019.00073

**Published:** 2019-04-05

**Authors:** Fabiane Sônego, Sophie Bouccara, Thomas Pons, Nicolas Lequeux, Anne Danckaert, Jean-Yves Tinevez, Israt S. Alam, Spencer L. Shorte, Régis Tournebize

**Affiliations:** ^1^UTechS Photonic BioImaging, C2RT, Institut Pasteur, Paris, France; ^2^Laboratoire de Physique et d'Etude des Matériaux, ESPCI Paris, CNRS UMR8213, PSL Research University, Université Pierre et Marie Curie, Sorbonne-Universités, Paris, France; ^3^UTechS Photonic BioImaging, C2RT, Unité Pathogénie Microbienne Moléculaire, Institut Pasteur, INSERM U1202, Paris, France

**Keywords:** quantum dot (QD), tumor, *in vivo* optical imaging, bioluminescence, fluorescence

## Abstract

Early detection of tumors is today a major challenge and requires sensitive imaging methodologies coupled with new efficient probes. *In vivo* optical bioluminescence imaging has been widely used in the field of preclinical oncology to visualize tumors and several cancer cell lines have been genetically modified to provide bioluminescence signals. However, the light emitted by the majority of commonly used luciferases is usually in the blue part of the visible spectrum, where tissue absorption is still very high, making deep tissue imaging non-optimal, and calling for optimized optical imaging methodologies. We have previously shown that red-shifting of bioluminescence signal by Fluorescence Unbound Excitation from Luminescence (FUEL) is a mean to increase bioluminescence signal sensitivity detection *in vivo*. Here, we applied FUEL to tumor detection in two different subcutaneous tumor models: the auto-luminescent human embryonic kidney (HEK293) cell line and the murine B16-F10 melanoma cell line previously transfected with a plasmid encoding the Luc2 firefly luciferase. Tumor size and bioluminescence were measured over time and tumor vascularization characterized. We then locally injected near infrared emitting Quantum Dots (NIR QDs) in the tumor site and observed a red-shifting of bioluminescence signal by (FUEL) indicating that FUEL could be used to allow deeper tumor detection in mice.

## Introduction

Imaging of physiological and pathological processes benefits from sensitive methodologies (Wehrl et al., 2014) and new imaging probes and methodologies are constantly evolving from the progress in preclinical research and important insights that it has yielded. Preclinical and small-animal imaging modalities allow longitudinal and multiparametric studies while reducing the number of animals used in the studies and thus comply with ethical guidelines. They include MRI, SPECT, and PET (Bernsen et al., 2014; Wehrl et al., 2014). Whilst MRI and nuclear imaging confer high resolution and sensitivity, respectively, the cost of these scanners and their maintenance represent major limitations in their use. By contrast, optical imaging is a widely used and low-cost methodology, also offering high sensitivity but also high throughput (O'Farrell et al., 2013).

Bioluminescence imaging has been widely used in the field of preclinical oncology. Several cell lines have been genetically modified to provide both *in vitro* and *in vivo* stable bioluminescence signals. In most cases, tumor cells are modified to express the enzyme luciferase and then a suitable substrate is added exogenously, which leads to the production of light in presence of oxygen and ATP (Marques and Esteves da Silva, 2009; O'Farrell et al., 2013). Recently, autonomous bioluminescent mammalian cell lines have been developed. These cell lines express both codon-optimized *Photorhabdus luminescens* luciferases coding genes and associated genes responsible for the production and recycling of aldehyde and FMNH_2_ co-substrates required for light emission. As a direct consequence, these cell lines do not require substrate addition to be luminescent and autonomously and constantly produce light (Close et al., 2010). In bioluminescence imaging, the photons are produced by the reporter luciferase enzymes present in the imaged subject (cells or animal). Because there is no non-specific bioluminescence signal, and unlike fluorescence, there is no need for an external excitation sources, the bioluminescence light is highly specific and detected with low background signals. However, the optical spectral region where common luciferases maximally emit is between 480 and 620 nm, where tissue absorption is maximum, highly limiting deep tissue bioluminescence imaging (Close et al., 2011; O'Farrell et al., 2013) while a range of wavelengths between 650 and 900 nm is more suitable for *in vivo* imaging (Frangioni, 2003). Several strategies have been developed in the last few years to overcome this limitation by red-shifting the emission in the well-adapted wavelength range where tissue absorption is minimal. One of the strategies adopted is the Bioluminescence Resonance Energy Transfer (BRET). BRET is a non-radiative process in which energy is transferred from a bioluminescent donor to a fluorescent acceptor that has been shown to be a powerful tool to evaluate protein-protein interaction (Wu and Brand, 1994; Pfleger and Eidne, 2006). Based on the principle of BRET, self-illuminated quantum dots (QDs) have been designed (Xiong et al., 2012). QDs are inorganic fluorescent nanocrystals that are ideal candidate as BRET acceptor due to their broad absorbance spectra, high absorbance cross sections, high fluorescence quantum yield, and their large Stokes shift in the near infrared (NIR) region (Michalet et al., 2005). In this context, carboxylate QDs coupled with amide luciferase and even functionalized with a RGD peptide have been developed for targeting *in vivo* cancer cells (So et al., 2006; Yuan et al., 2015; Kamkaew et al., 2016; Trapiella-Alfonso et al., 2018).

Recently, we reported Fluorescence by Unbound Excitation from Luminescence (FUEL) as a mean to red-shift bioluminescence emission without requiring extremely close contact between donor and acceptor like in BRET. FUEL is defined as a radiative transfer between a bioluminescent source exciting nearby fluorophore (Dragavon et al., 2012; Holland et al., 2014). We have hypothesized that FUEL could be a useful tool for enhancing the detection of bioluminescent tumors in preclinical imaging due to two main advantages. Firstly, luciferase does not need to be grafted to the nanoparticles. This would allow the use of smaller diameter nanoparticles, likely to have superior pharmacokinetic properties in comparison to coupled larger nanoparticles (Choi et al., 2007; Perrault et al., 2009). Secondly, FUEL is a relevant mean to increase the signal sensitivity in targeted tissue because QDs red emission is spatially correlated with the tumor bioluminescence signal and results to be a marker of proximity. However, this phenomenon would be only applicable to preclinical tumor imaging, as it requires a bioluminescent tumor cell line as source of excitation for QDs.

In this study, we focused on two different *in vivo* subcutaneous bioluminescent tumor models to investigate the feasibility of FUEL in detecting tumors. The first model was induced by bioluminescent B16-F10 tumor cells expressing firefly luciferase (Albanesi et al., 2012, 2013; Danciu et al., 2013). These cells will be referred here as B16-Luc2. The second tumor model established here was a bioluminescent HEK293 model, a human embryonic kidney cell line expressing the lux operon from bacteria and will hereon be referred as HEK-Lux. This cell type expresses both the luciferase and enzymes required for the production of the substrate, and therefore does not require further administration of substrate to be autonomously bioluminescent (Close et al., 2010). Using these two models, we present and quantify the first *in vivo* FUEL experiments using near-infrared emitting quantum dots to achieve a red-shifting emission of the subcutaneous tumors in mice.

## Methods

### Cell Lines Culture

The autobioluminescent HEK293 cells with the luxCDABE operon (HEK-Lux) cells were kindly provided by 490 BioTech (Tennessee, USA) (Xu et al., 2014). These cells were cultured at 37°C and 5% CO_2_ in DMEM with Glutamax and Pyruvate (Life technologies) supplemented with 10% heat-inactivated fetal bovine serum (FBS, Gibco), 1% of non-essential amino acids (Sigma), 1% penicillin/streptomycin (Life technologies), and 100 μg/mL G418 (Sigma). The experiments were performed with cells at passage 20–22.

Non-autobioluminescent HEK293 cells were cultured in the same medium as HEK-Lux cells, but in the absence of antibiotic G418. At confluence, cells were rinsed with phosphate buffered saline without Ca^2+^ and Mg^2+^ (PBS, Gibco) and harvested with 0.05% trypsin-EDTA (Gibco). Cells were used at passage 9.

The melanoma cell line B16-F10, expressing Luc2 (B16-Luc2) was kindly provided by the group of Pierre Bruhns (Institut Pasteur, Paris). The cells were cultured in RPMI 1640 with glutamine and Hepes (Gibco) supplemented with 10% heat-inactivated FBS and 1% penicillin/streptomycin. At maximum 50% of confluence, cells were rinsed with PBS and harvested with 0.05% trypsin-EDTA. The experiments were performed with cells at passages between 6 and 16.

The emission spectra of the HEK-Lux and B16-Luc2 cells were determined using 2 × 10^5^ cells suspended in 0.1 mL of appropriated medium. One day prior to imaging, cells were seeded in a 96-well clear bottom black plate (Nunc) and incubated overnight at 37°C and 5% CO_2_. The medium was gently removed from the wells and replaced with fresh medium prior to image acquisition. For B16-Luc2 cells, the substrate D-luciferin (Perkin Elmer) was added to the cells (150 μg/mL in 0.01 mL). Bioluminescence images were acquired with an IVIS Spectrum system, using 20 nm bandpass emission filters and OPEN mode (exposure time of 180 s for HEK-Lux cells and 30 s for B16-Luc2 cells).

### Mice and Ethics Statement

Female nude mice (Rj:NMRI-nu) (7 weeks-old) were obtained from Janvier Laboratories (France). All protocols involving animal experiments were approved and carried out in accordance with the ethical guidelines of Institut Pasteur, Paris, and approved by the Comité d'éthique de l'Institut Pasteur (CETEA comité d'éthique en experimentation animale n°89) under the protocol license number: 2014-0055. The mice were housed in the Biosafety Level 2+ animal facility of Institut Pasteur. All mice had free access to food and water and were under controlled light/dark cycle, temperature and humidity. Animals were handled with regard for alleviation of suffering. Animals were anesthetized using isoflurane, and euthanized with CO_2_.

### Induction of Subcutaneous Tumors

#### HEK-Lux and Non-bioluminescent HEK Models

Each tumor was induced by subcutaneous (s.c.) administration of 0.1 mL of 5 × 10^6^ cells (suspended in medium without FBS) and basement membrane matrix growth factor reduced (matrigel Corning), (25:75, v/v).

#### B16-Luc2 Model

Each tumor was induced by s.c. administration of 0.1 mL of 8 × 10^4^ cells (suspended in medium without FBS) and basement membrane matrix growth factor reduced (matrigel, Corning), (20:80, v/v).

For all cell lines, culture medium was replaced with fresh medium 1 day prior to the subcutaneous injection.

Two ventral tumors were induced in each mouse. The mice were anesthetized with 2% isoflurane gas prior to the injection of the tumor cells. Cells were first administered subcutaneously on the left side and then on the right side of the mice. All the results shown here represent measurements taken for the left tumor of each mouse. Tumor growth was monitored by caliper measurement and tumor volume determined as previously described; volume = [(width/2)^2^ × length] (Ho et al., 2004).

### Near Infra-Red (NIR) QDs

**Figure 1:**
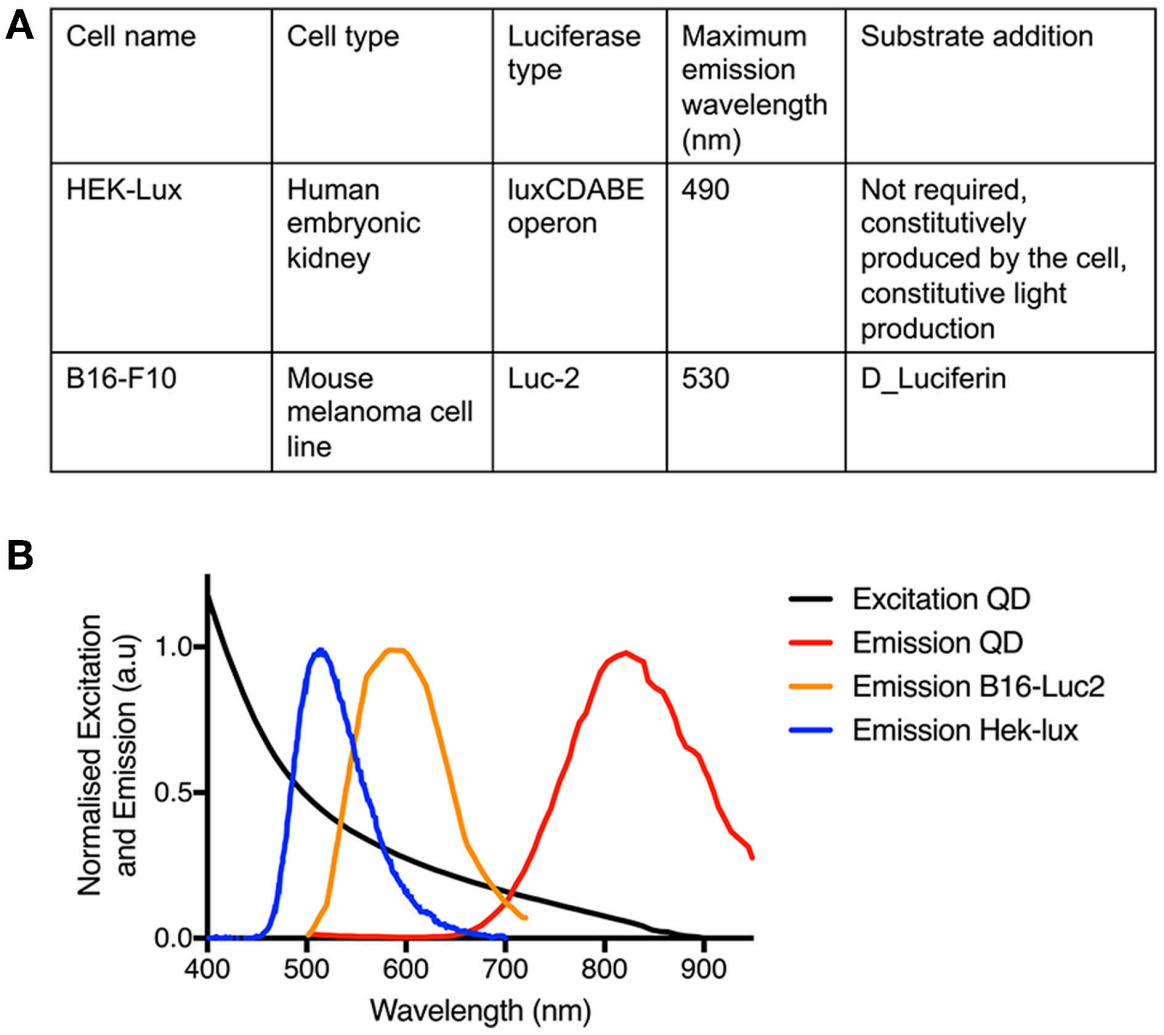
Characteristics of the materials used. **(A)** Table presenting the features and properties of the two cell lines. **(B)** Normalized absorption spectrum of the NIR QDs and normalized emission spectra of the NIR QDs, the HEK-Lux cells, and the B16-Luc2 cells.

NIR QDs were synthesized as previously described (Bouccara et al., 2014) and water-solubilized as described in Tasso et al. (2015). Briefly, the size of the QDs is 3–5 nm in diameter as measured by transmission electron microscopy (Bouccara et al., 2014; Trapiella-Alfonso et al., 2018). After solubilization with the zwitterionic copolymer, their hydrodynamic radius is about 9 nm and their zeta potential is slightly negative (typically ≈ −10 mV) (Trapiella-Alfonso et al., 2018). Their photoluminescence emission maximum is at 810 nm (Figure 1) and their photoluminescence quantum yield at ~15–25% when measured using indocyanine green as a standard (Φ = 13% in DMSO). These QDs have an excellent colloidal stability in biological buffers and show no aggregation and limited non-specific adsorption in albumin solutions and whole serum, even after 48 h of incubation (Debayle et al., in review).

NIR QDs were diluted in PBS to provide the desired concentration. Absorption and emission spectra of a 0.1 μM solution were determined using IVIS Spectrum.

### *In vivo* Bioluminescence and Fluorescence Imaging

Bioluminescence and fluorescence imaging were performed using an IVIS Spectrum system (Perkin Elmer). Unless specified elsewhere, mice bearing the autobioluminescent HEK-Lux tumors were anesthetized with 2% isoflurane gas and typically imaged with (840 nm) and without emission filter (total light output—open filter) for 300 s. Mice bearing the bioluminescent B16-Luc2 tumors were intraperitoneally (i.p.) administered with the substrate D-luciferin (0.75 mg/mouse, Perkin Elmer) 11 min prior to bioluminescence imaging. This time point was chosen to allow a comparison between different mice and because it corresponds to the D-luciferin peak bioavailability. Mice were anesthetized with 2% isoflurane gas immediately after the administration of D-luciferin and maintained under anesthesia until the end of the image acquisition. Bioluminescence images were acquired in the open mode or with the 840 nm filter for 180, 60, or 3 s, as specified in figures legends. Fluorescence images were also acquired using IVIS Spectrum system (excitation filter 430 nm and emission filter 840 ± 20 nm). Living Image software (Perkin Elmer) was used to define and analyze the light emission in the regions of interest (ROIs).

#### Angiosense 750EX

The fluorescent vascular agent Angiosense 750EX (Perkin Elmer) was administered intravenously (i.v.) (2 nmol/0.1 mL) in mice bearing HEK-Lux or B16-Luc2 tumors, 22–30 or 7–9 days post-tumor cells injection, respectively. Mice were anesthetized with 2% isoflurane gas prior to the image acquisition. The vascularization of the tumors was evaluated 24 h post-Angiosense 750EX administration using the IVIS Spectrum system. Fluorescent images were acquired with 745 nm excitation filter and 800 nm emission filter, with the auto option selected as time of exposure.

#### NIR QDs

Fluorescent images using IVIS Spectrum were acquired prior and after NIR QDs intratumoral administration *in vivo* with 0.1 s of exposure time, and 430 and 840 nm as excitation and emission filters, respectively.

### Dextran- Fluorescein Isothiocyanate (FITC)

High molecular weight dextran-FITC (500 KMW, Molecular Probes) was injected i.v. *via* the retro-orbital sinus (0.5 mg/0.1 mL) in mice bearing HEK-Lux or B16-Luc2 tumors. Harvested tumors were fixed in 4% paraformaldehyde (EMC) for 3–5 h at room temperature, depending on the tumor volume, followed by aldehydes quenching by 1 h incubation in 100 mM glycine (Sigma-Aldrich). Tumors were then incubated in 15% sucrose (Sigma-Aldrich) at 4°C overnight and in 30% sucrose at 4°C for ~24 h before embedding in Shandon Cryomatrix (Thermo Fischer) and freezing using isopentanol. Fifty micrometer sections cut using cryostat (CM3050 S, Leica) were stained with DAPI and imaged using an automated spinning disk microscope CellVoyager1000 (Yokogawa Electrics, Japan). The sections were left overnight at room temperature before being stained with DAPI.

### FUEL Experiments

#### *In vitro* FUEL

B16-Luc2, HEK-Lux, and HEK non-bioluminescent cells (2 × 10^5^, 0.1 mL of appropriated medium) were seeded in a 96-well clear bottom black plate (Nunc) 1 day prior to the experiment and incubated at 37°C and 5% CO_2_. On the day of the experiment, the medium was removed and a fresh medium with or without NIR QDs (450 μM in 0.01 mL) was added to the well. Each cell type was cultured with the same medium used for the cell culture. HEK non-bioluminescent cell type was used in this experiment as a negative control for HEK-Lux cells. For B16-Luc2 cells, the substrate D-luciferin was added to the wells (150 μg/mL in 0.01 mL), and the absence of the substrate in the well was used as a negative control for this cell type. Bioluminescence images were acquired with both 840 nm and open filter (exposure time of 300 s for HEK cells and 180 s for B16-Luc2 cells). Fluorescence images were also acquired (excitation 430 nm and emission 840 nm, 1 s as exposure time).

#### Experiments With Mice Bearing B16-Luc2 Tumors

In order to evaluate the bioluminescence signal emitted at 840 nm before the administration of NIR QDs, D-luciferin (0.75 mg/mouse, i.p.) was administered in mice bearing B16-Luc2 tumors 11 min prior to the image acquisition (180 s as exposure time). After 1 h, bioluminescent images were acquired again to determine the basal bioluminescent signal at 840 nm. Next, 0.5 nmol (0.04 mL) NIR QDs were administered into the left tumor and 0.04 mL PBS into the right tumor. Fluorescence images were acquired (excitation 430 nm/emission 840 nm, 0.1 s) prior and post-NIR QDs intratumoral administration. D-luciferin was then administered 11 min prior to the bioluminescence imaging acquisition with a 840 nm and open filter for 180 and 3 s, respectively.

Experiments were also performed to evaluate the possible effect of NIR QDs without a bioluminescence source. For this control, NIR QDs were injected in the left tumor and PBS was injected in the right tumor of the mice, without previous administration of D-luciferin. Both bioluminescence and fluorescence images were acquired, using the same emission and excitation filters and exposure time.

#### Experiments With Mice Bearing HEK-Lux Tumors

Bioluminescence images at 840 nm and open filter (300 s of exposure time) were acquired prior and post-injection of 0.5 nmol (0.04 mL) of NIR QDs in the left tumor and 0.04 mL of PBS in the right tumor of mice bearing the autobioluminescent HEK-Lux tumors. Fluorescence images were acquired (excitation 430 nm and emission 840 nm, 0.1 s) prior and post-NIR QDs intratumoral administration.

### Statistics

The number of experimental repeats and animals used for each experiment are noted in the figure legends. When compared, B16-Luc2 and HEK-Lux tumors results were analyzed via Mann-Whitney test or Student's *t*-test after being assessed for normality of sample distribution. For the statistical analyses, the results from *in vitro* experiments were analyzed after normalization by strictly standardized mean difference (SSMD) test as previously described (Mellouk et al., 2014). Statistical analyses and graphs plotting were performed using Prism 6.0 (GraphPad Software, USA). *P*-values of ^*^p < 0.05 and ^**^p < 0.001 were used.

## Results

### Characterization of Tumor Models Reveals Marked Differences in Bioluminescence Emission and Growth Dynamics but Shows Similar Vascularization

In order to investigate the ability of FUEL to enhance the detection of tumors *in vivo*, we used two distinct bioluminescent preclinical subcutaneous tumor models in nude mice: murine B16-Luc2 melanoma tumors previously described (Albanesi et al., 2013) and the human HEK 293 tumor model, adapted from the model described by Ho et al. (2004). Figure 1 summarizes the different properties of the cell lines used and represent the optical spectra of the QDs and bioluminescent tumor cell lines.

**Figure 2:**
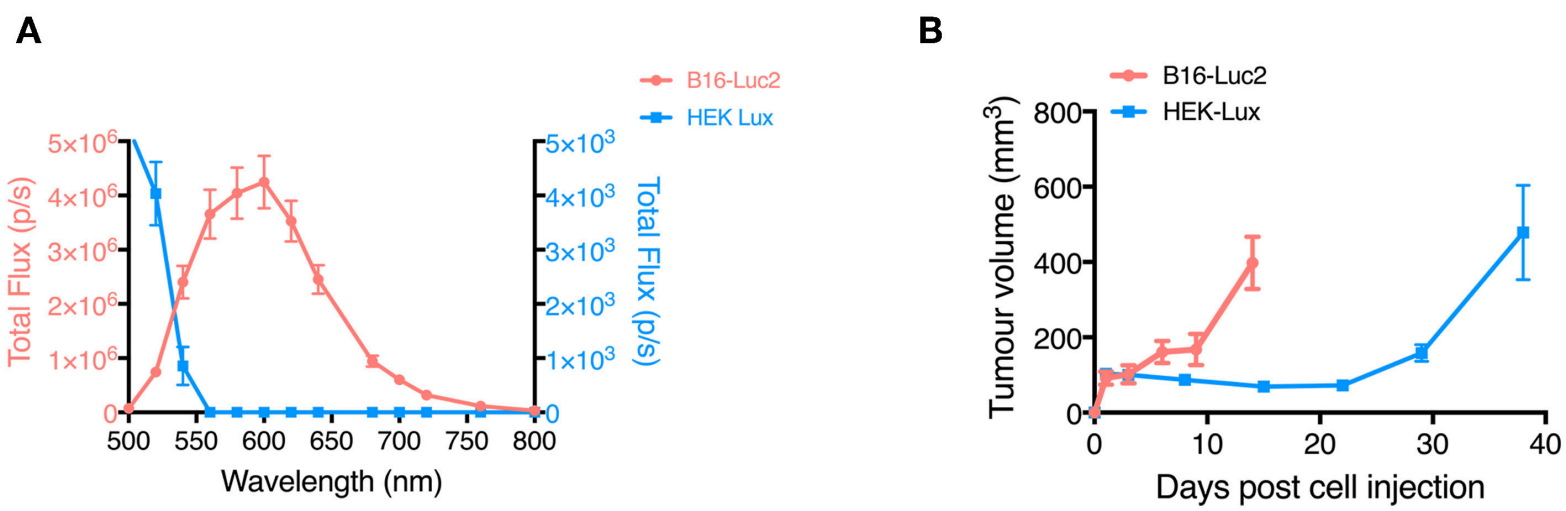
Characterization of emission spectra of B16-Luc2 and HEK-Lux cells and tumor growth curves. **(A)** Emission spectrum of B16-Luc2 and HEK-Lux cells. Bioluminescence images were acquired from 500 to 840 nm for 30 s (B16-Luc2) or 180 s (HEK-Lux). Results are expressed as total flux (photons/s) in the ROI, *n* = 3. **(B)** Tumor growth of B16-Luc2 (8 × 10^4^, 0.1 mL) and HEK-Lux (5 × 10^6^) cells over time, following subcutaneous injection in nude mice on the right and left sides. Results are representative of 4 independent experiments and represent the left tumor volume measured with a caliper, *n* = 5. Data shown are means ± SEM.

Firstly, we measured the emission spectrum for each of the tumoral cell types on the imaging system used and observed an emission peak at 600 nm for B16-Luc2, while for HEK-Lux the peak was at 500 nm (Figure 2A). It is noteworthy that the B16-Luc2 cells emit a stronger bioluminescent signal when compared to an equal number of HEK-Lux cells. B16-Luc2 cells also showed higher *in vivo* proliferation than HEK-Lux cells. While 8 × 10^4^ B16-Luc2 cells induced the formation of 400 mm^3^ tumors in 14 days, 5 × 10^6^ HEK-Lux cells were necessary to induce similar tumor sizes in more than 30 days (Figure 2B).

**Figure 3:**
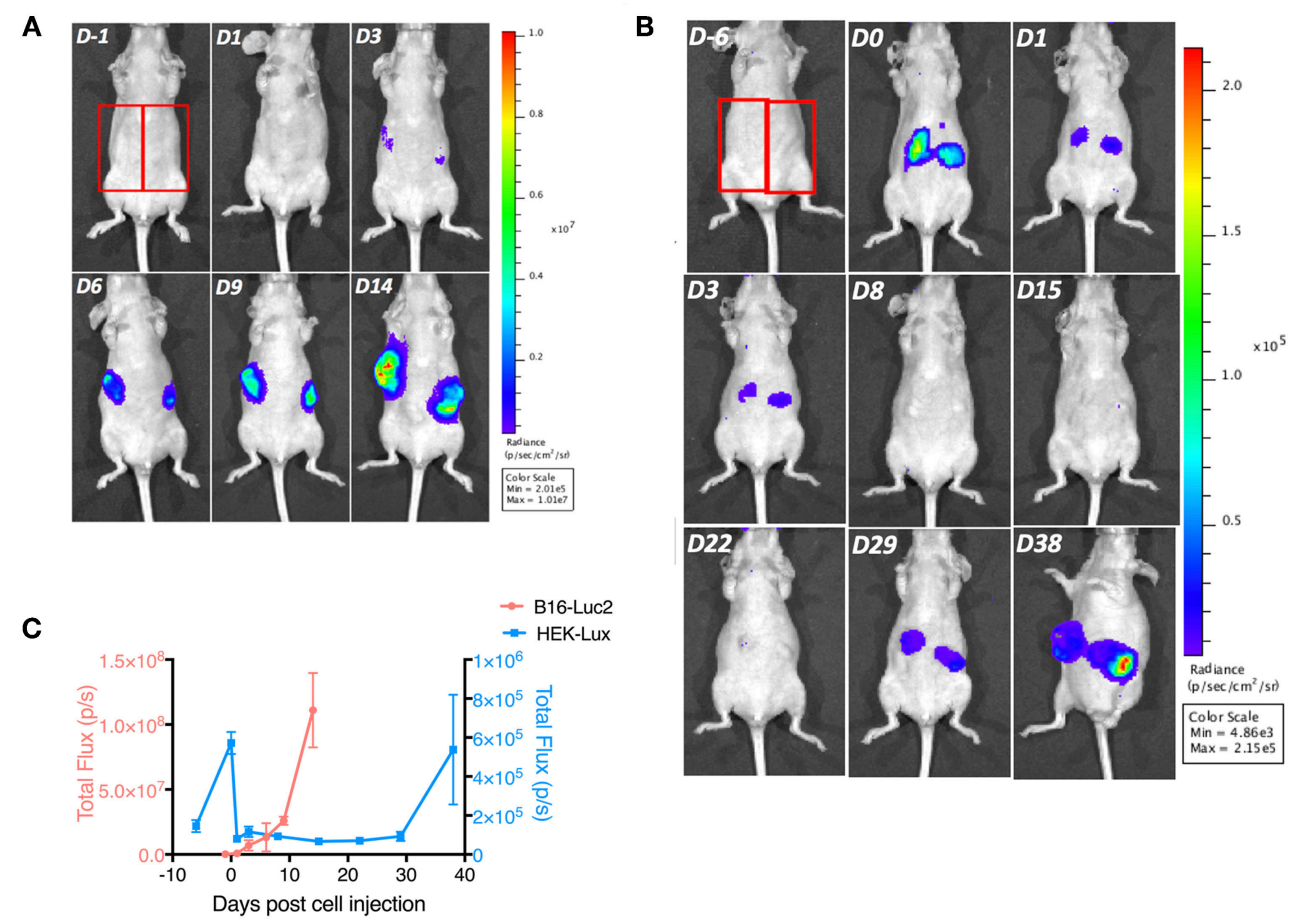
Tumor bioluminescence signal evolution imaging over time. **(A)** B16-Luc2 cells (8 × 10^4^, 0.1 mL) were subcutaneously administered in nude mice. Mice were imaged 1 day prior and 1, 3, 6, 9, and 14 days post-administration of B16-Luc2 cells, *n* = 5. **(B)** HEK-Lux cells (5 × 10^6^, 0.1 mL) were subcutaneously adminstered in nude mice. Mice were imaged 6 days prior and 0, 1, 3, 8, 15, 22, 29, and 38 days post-administration of HEK-Lux cells, *n* = 6. **(C)** Bioluminescence signal quantitation of B16-Luc2- and HEK-Lux-induced tumors. Red rectangles in 2A and 2B show the ROI used for quantification. Results express the total flux (photons/s) in the ROI of the left tumor of the mice. These results are representative of 4 independent experiments.

We also acquired bioluminescence images of tumors over time, and observed that similar to the growth in tumor volume, the bioluminescence signal intensity of B16-Luc2 tumors was detectable as early as 3 days post-injection and increased over time to reach ~10^8^ photons emitted/s per tumor on day 14 (Figures 3A,C). In contrast, HEK-Lux cells produced significantly much less light with a different kinetic. Although HEK-Lux cells emitted a detectable bioluminescence signal immediately after the subcutaneous injection, this signal disappeared on day 1. The signal stayed low until day 29, when it started to increase again, reaching a maximum of 10^5^ photons/s per tumor on day 38 (Figures 3B,C). Interestingly, the signal increase correlated with the development of the tumor, as assessed by an increase in tumor volume, suggesting that the cells had a latency time before growing and emitting higher bioluminescence signal. Altogether, these observations show that the two tumor models have markedly different dynamics of the growth curves and that the B16-Luc2 tumors emit 1,000 times more light using an open filter for detection than the HEK-Lux.

**Figure 4:**
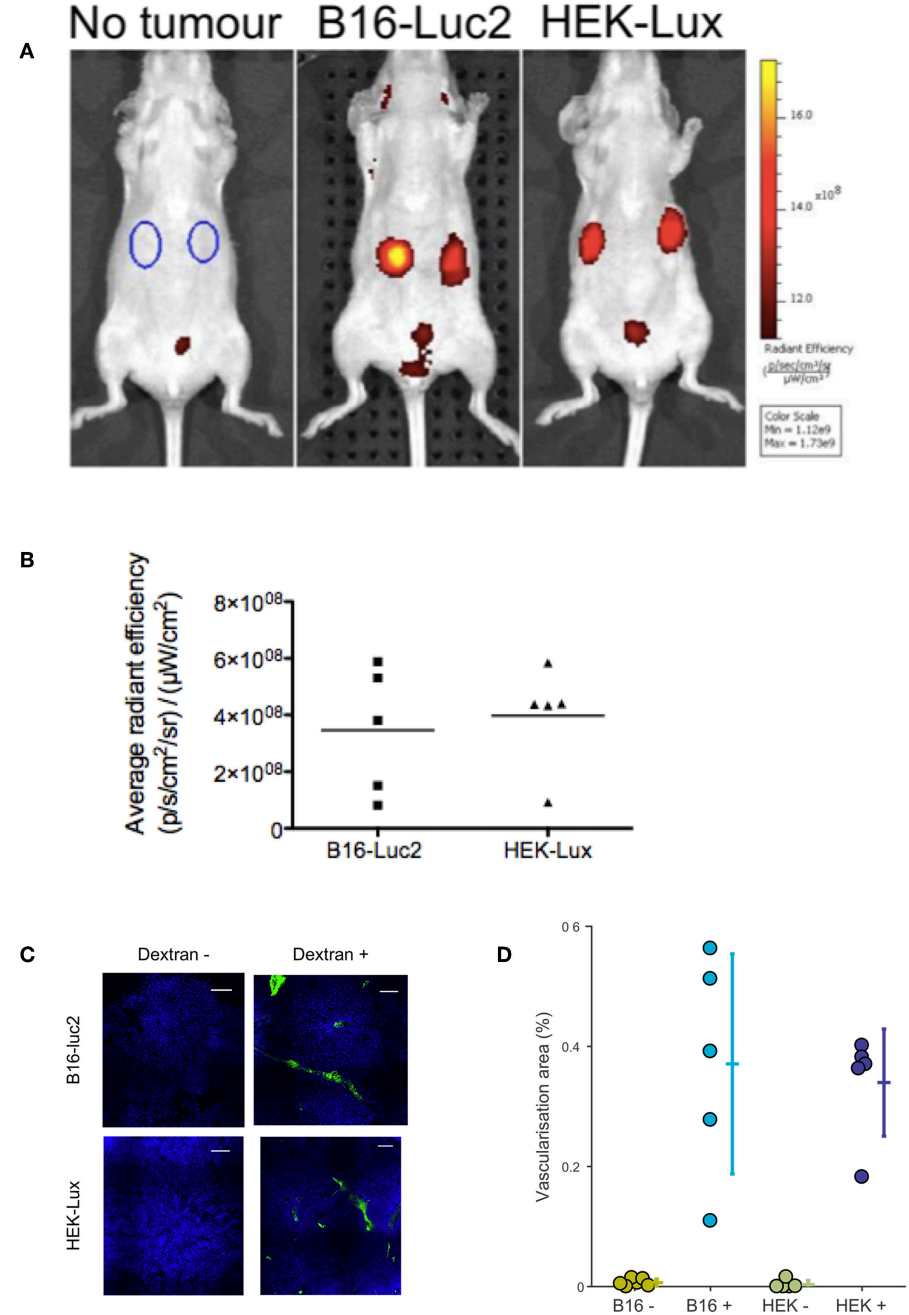
*In vivo* evaluation of tumor vascularization. **(A)** B16-Luc2 cells (8 × 10^4^, 0.1 mL), HEK-Lux (5 × 10^6^, 0.1 mL) were subcutaneously administered in nude mice. Angiosense 750EX (2 nmol, 0.1 mL) was intravenously administered between 7 and 9 days after B16-Luc2 injection or between 22 and 30 days post-HEK-Lux cells injection Images were acquired 24 h after. **(B)** Fluorescence signal quantitation of Angiosense accumulation in B16-Luc2- and HEK-Lux-induced tumors. ROIs were determined as shown in the first image of Figure 3A. Results express the difference between the average radiant efficiency in the ROI of the left tumor of the mice with tumor and the arithmetic mean of the average radiant efficiency in the ROI of the left side in mice without tumor, (*n* = 4 control group and *n* = 5 for the tumor bearing groups). **(C)** Visualization of tumor vascularization using high molecular weight dextran-FITC (500 KMW). Images correspond to a section in the tumors at 50% depth. Contrast and brightness in both channels have been adjusted with an identical color scale across the four images. Scale bars: 100 μm. **(D)** Area of vascularization, defined as the percentage of the tumor area labeled by dextran at 0, 25, 50, 75, and 100% tumor depth. The area of vascularization was extracted using an identical threshold over all images.

We additionally investigated the vascularization of both tumors using the vascular agent Angiosense 750EX. Fluorescence images acquired 24 h post-Angiosense administration indicated similar accumulation of the probe in both B16-Luc2 and HEK-Lux-induced tumors (Figures 4A,B). Mice not bearing tumors were used as control, and did not show fluorescence signal in the upper abdomen. The fluorescence signal observed in the lower abdomen, in both control and tumor-bearing mice, is likely associated with the renal excretion of the probe. In order to investigate the vascularization at microscopic levels, we have administrated high molecular weight dextran labeled with FITC i.v. Corroborating the results *in vivo*, histological sections suggest that the vascularization is similar in both tumor models (Figures 4C,D).

### FUEL Enables Enhanced Detection of Tumors

**Figure 5:**
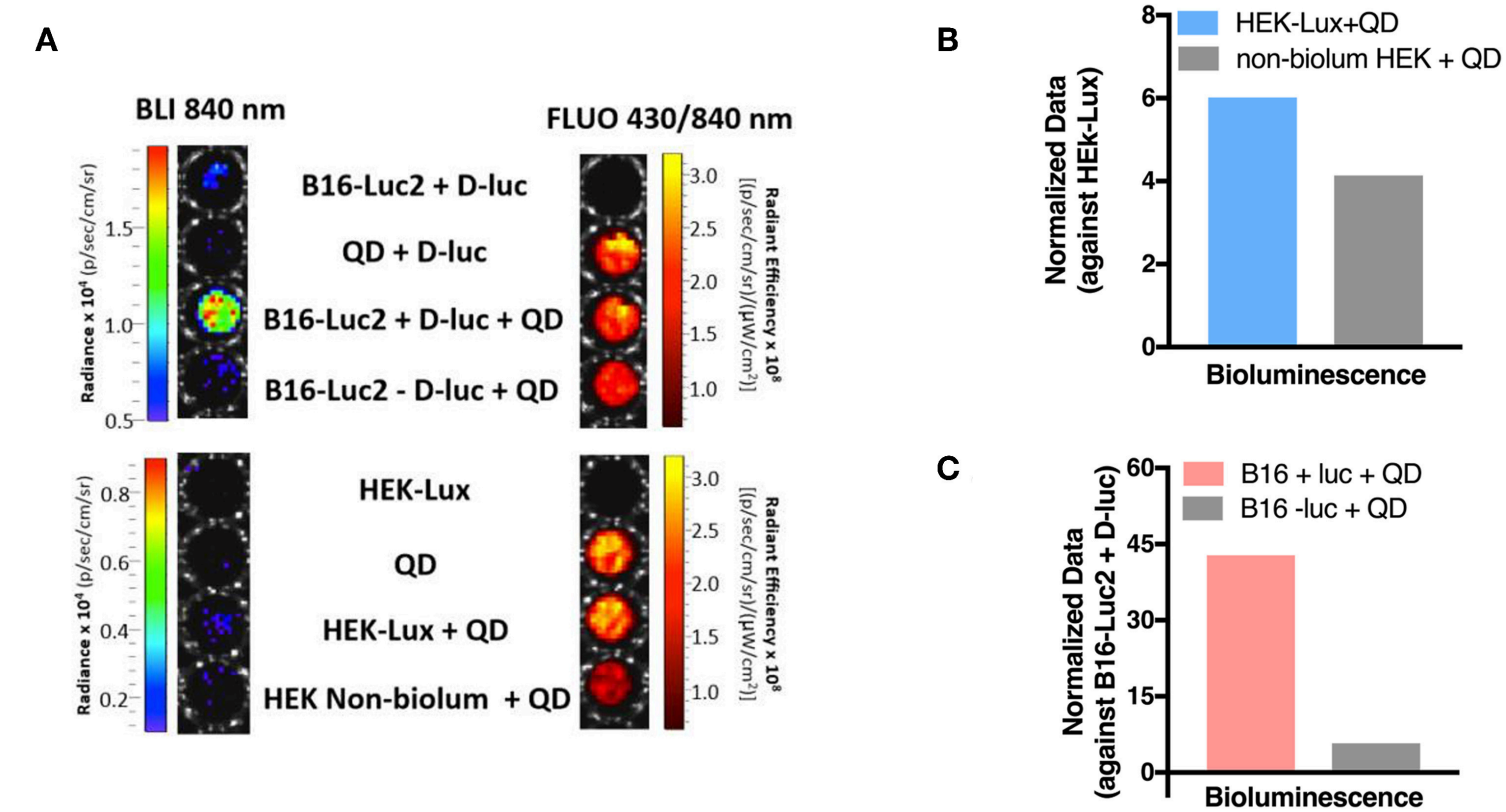
*In vitro* investigation of FUEL with NIR QDs. **(A)** Bioluminescence (840 nm, exposure time of 60 s (B16-Luc 2 cells) and 180 s (HEK-Lux cells), as well as fluorescence images (excitation 430 nm, emission 840 nm, and exposure time of 1 s). **(B)** Quantitation of bioluminescence signal emitted at 840 nm. Results are expressed as normalized SSMD values for B16-Luc2 cells (B16-Luc2 cells + D-luciferin used as control) or **(C)** HEK-Lux (or non-bioluminescent HEK used as control). *n* = 8 (except for HEK-Lux + QD – *n* = 6).

FUEL efficiency depends on the overlap between the emission spectrum of the bioluminescent source and the excitation spectrum of the acceptor fluorophore. NIR QDs have a broad and continuous decreasing excitation spectrum from UV to 800 nm, as illustrated in Figure 1B. This spectrum suggests that both B16-Luc2 (with an emission peak wavelength centered at around 600 nm) and HEK-Lux bioluminescence signal (with an emission peak wavelength centered at around 500 nm) are suitable for the excitation of NIR QDs. Additionally, emission spectrum indicates a maximum emission at around 840 nm. Based on these spectra, we first investigated the presence of FUEL with both B16-Luc2 and HEK-Lux *in vitro*. The incubation of B16-Luc2 cells with NIR QDs significantly increased the bioluminescence signal at 840 nm as compared to cells alone and B16-Luc2 incubated with NIR QDs but in the absence D-luciferin (Figure 5A). The presence of the NIR QDs in the specified wells was confirmed by the fluorescence image (Figure 5A). Normalized SSMD values classified the FUEL phenomenon extremely strong as compared to the controls (Figures 5B,C). HEK-Lux cells, which emit weaker bioluminescence signals, also showed an increase in the intensity of bioluminescence at 840 nm in the presence of NIR QDs. The statistical analyses using SSMD normalization indicate a very strong difference between HEK-Lux cells incubated with NIR QDs and controls (HEK-Lux cells alone, and non-bioluminescent HEK cells incubated with NIR QDs) (Figure 5B). It is important to mention that the scales for B16-Luc2 and HEK-Lux are different due to the intensity of the bioluminescence emitted by each cell types.

**Figure 6:**
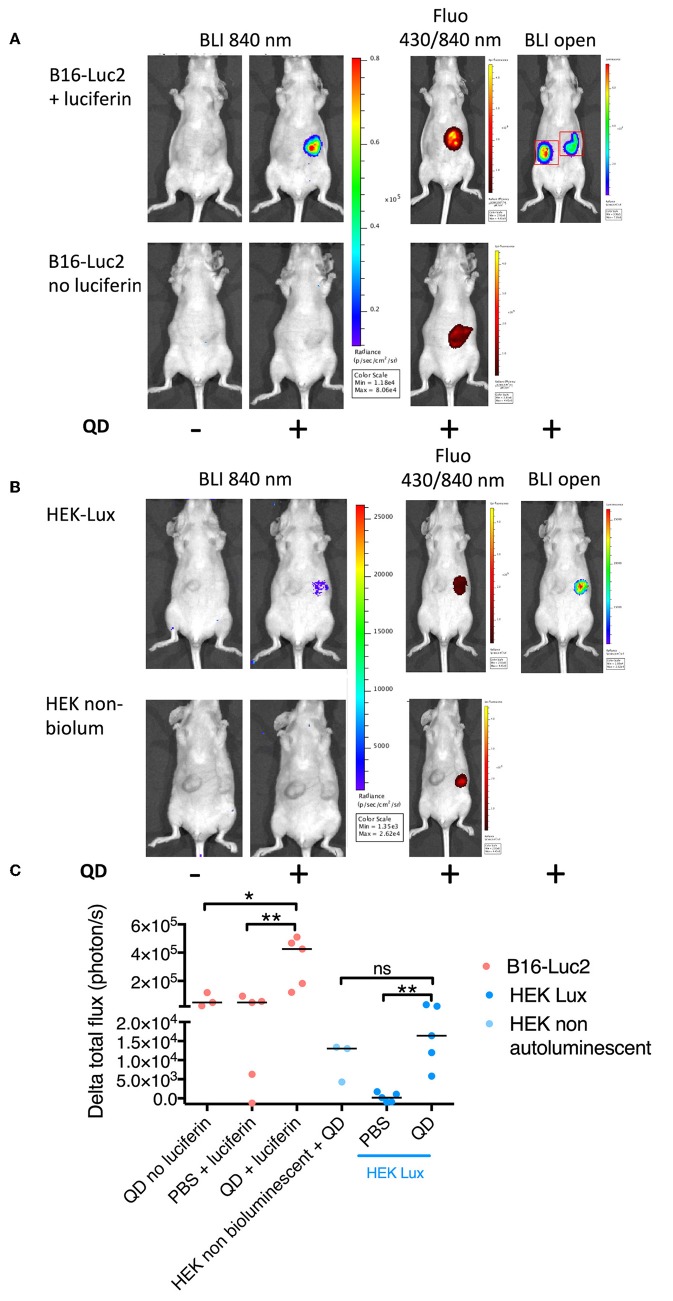
*In vivo* evaluation of FUEL. Bioluminescence imaging at 840 nm of B16-Luc2 **(A)** or HEK-Lux **(B)** tumors prior (left image) or after quantum dots injection in the right tumor (2nd image left). Fluorescence images and bioluminescence in open mode are shown on the right. 840 nm bioluminescene images of control without luciferase for B16-Luc2 Cells **(A)** or non-bioluminescent HEK cells **(B)** are shown in the second row. **(C)** Quantitation of FUEL phenomenon. ROIs were determined as shown in the image of Figure 5A. Results express the difference between total flux (photons/s) in the ROI of the left tumor of the mice post-NIR QDs injection and prior to the NIR QDs injection, *n* = 3 (negative control groups), *n* = 6 (B16-Luc2), and *n* = 4 (HEK-Lux). *p* < *0.05* was considered as significant: *p < *0.05* and **p < *0.001*.

We next investigated the ability of FUEL to red-shift tumor emission at the NIR QDs wavelength, enhancing the detection of tumor at red range wavelengths. Mice bearing B16-Luc2 tumors were imaged after the i.p. administration of D-luciferin to evaluate the background signal at 840 nm (–QD/+luciferin) (Figure 6A). After the intratumoral injection of NIR QDs (+QDs/+luciferin), we observed a drastic increase in the bioluminescence signal at 840 nm, confirming the presence of FUEL and its ability to enhance tumor detection at 840 nm by red shifting the light emission (Figures 6A,C). Fluorescence imaging confirmed the presence of NIR QDs in the tumor sites and bioluminescence imaging in open filter shows that both right and left tumors were bioluminescent upon the administration of D-luciferin. No signal was observed in the absence of the substrate (–QD/–luciferin and +QD/–luciferin).

FUEL efficiency was also investigated in HEK-Lux-induced tumor model. Bioluminescence signal at 840 nm post-intratumoral administration of NIR QDs was stronger than pre-injection (–QD/HEK-Lux vs. +QD/HEK-Lux, Figures 6B,C). NIR QDs administered into non-bioluminescent HEK293 tumors showed bioluminescence signal statistically similar to HEK-Lux tumors with NIR QD.

In summary, we have shown that both bioluminescent tumor models undergo a red shifting in their emission via FUEL, where the red-shifting emission strongly depends on the optical emission properties of the tumors and the quantum yield of the near-infrared emitting fluorescent probe.

## Discussion

The development of new techniques for detecting tumors in an accurate and simple way is vital to support the search for new therapies in oncology. In this study, we used two different bioluminescent tumor models to demonstrate for the first time, that the FUEL process can be used *in vivo* to red-shift bioluminescence tumor emission and enhance the detection of tumors in the infrared region.

Herein, we established two murine models of tumors to investigate FUEL. One of the models was xenogeneic and made use of human (HEK-Lux) cells, a constitutively bioluminescent cell type (Close et al., 2010). The second model was syngeneic, induced by B16-Luc2, a murine melanoma cell type expressing the enzyme luciferase frequently used in preclinical oncology (Overwijk and Restifo, 2001). While B16-Luc2 tumor growth and their bioluminescence signal showed the same profile, HEK-Lux cells initially presented a high bioluminescence activity immediately after the subcutaneous injection before showing a marked decrease of this activity the following day. We believe that these cells needed to adapt to the new environment, resulting in undetectable light production, before propagating and forming the solid tumor. After this latency period, the tumors reached the maximal volume that corresponded with the second peak of bioluminescence emission.

The developed models show different behaviors and were used as a proof of concept of the feasibility of tumor detection using FUEL *in vivo*, as well as to identify the critical conditions allowing FUEL to occur. Each of the two models has advantages and disadvantages with regard to FUEL applications. HEK-Lux cells have the enormous advantage of being autobioluminescent due to their constitutive expression of the bacterial lux operon thus enabling convenient image acquisition without having to consider the biodistribution kinetics of exogenously added substrate *in vitro* or *in vivo* (Close et al., 2010) unlike for the B16-Luc2 cells (Albanesi et al., 2012). This required substrate injection is an important limitation since the time between substrate injection and imaging needs to be strictly controlled to obtain quantitative reproducible and comparable data, mainly when acquiring images using different emission filters before and after the injection of NIR QDs. In addition, melanin production by the B16-Luc2 cells might be a concern for this type of imaging. However, we observed that melanin expression becomes significant only 2 weeks after subcutaneous injection, after we performed our experiments, and that these cells are indeed suited for FUEL imaging (Figure 6).

FUEL is a phenomenon that allows the red shifting of the light, enhancing the detection of bioluminescent tumors because of the reduction of tissue absorption and scattering of blue/green light. One of the requirements for effective FUEL is that the fluorophore should have a large Stokes shift, determining the requirement of an ideal bioluminescent emitting source at ~500 nm (Dragavon et al., 2012; Holland et al., 2014). In this context, the wavelength of the maximal bioluminescence emission peak of HEK-Lux cells would be another advantage over B16-Luc2 cells regarding FUEL. Indeed, HEK-Lux cells emit luminescence at a maximum peak of 490 nm (Close et al., 2010). By contrast, B16-Luc2 cells have a maximum emission peak at 600 nm. In our case, we were still able to observe FUEL with B16-Luc2 because we used NIR QDs, which have a large absorption range. Furthermore, B16-Luc2 cells showed much stronger bioluminescence signal intensity in comparison to HEK-Lux cells, requiring shorter exposure times during imaging and overall higher FUEL efficiency. Our results show that even if HEK-Lux cells have a more appropriate maximum emission wavelength to excite NIR QDs than B16-Luc cells, due to their lower luminescence intensity, the red-shifting emission is not optimal. Indeed, if we focus on the maximum emission wavelength of both cell types, 500 and 600 nm for HEK-Lux cells and B16-Luc2 cells, respectively, NIR QDs absorb 4 times less at 600 nm than at 500 nm (Figure 1B). However, for an identical number of cells, at their maximum wavelength, the B16-Luc2 cells are about 800 times brighter at 600 nm than the HEK-Lux cells are at 500 nm (when the same number of cells are compared). At 500 nm, B16-Luc2 emission is still 14 times higher than HEK-Lux cells emission (Figure 2A). These results highlight the fact that FUEL efficiency is controlled by a combination of both luminescence spectrum and intensity as well as acceptor absorbance properties. Having theoretical model of the interactions governing FUEL *in vivo* would help design such best possible combinations. Nevertheless, NIR QDs have many advantages for FUEL applications; namely high excitation coefficient and photoluminescence quantum yield. Moreover, this specific type of NIR QD has been shown to provide a lower *in vivo* toxicity compared to classical NIR QDs mainly because they are not composed of heavy metals (Pons et al., 2010). In addition, FUEL efficiency also depends on the imaging conditions. Indeed, the emission filters used in this study have a narrow 20 nm bandwidth, which limits the imaging of the red-shifted emission photons. Using larger emission filter bandwidth or long pass emission filter should significantly improve the FUEL efficiency.

Our results suggest that FUEL enables red shifting emission of bioluminescent tumors to the near infrared region, that is a major advantage for deep and metastatic tumor detection. As an optical method, FUEL has the significant advantage of requiring affordable imaging systems and facilities (O'Farrell et al., 2013) that are extremely valuable in preclinical research. However, the experimental conditions of FUEL phenomenon for detecting tumors warrants some improvement and characterization to be fully suitable for enhanced detection of deeper tumors *in vivo*. Several factors mainly need to be taken into account: the biodistribution of the QDs within the xenograft, considering the tumor heterogeneity, and the fact that the tumor micro-environment could affect both luciferase enzymatic efficiency and fluorophore quantum yield, and consequently overall FUEL efficiency. The enhanced permeability retention (EPR) effect exhibited by tumors as a result of leaky vasculature, could favor the retention of nanoparticles (Blanco et al., 2015). An effective EPR effect is strongly dependent on the size of the nanoparticles, their surface chemistry and the type of tumor. For instance, the accumulation and distribution of micelles of various sizes was not substantially affected by the size in a colon adenocarcinoma (C26) model, while micelles size prove to be important in a human pancreatic adenocarcinoma (BxPC3) (Cabral et al., 2011). Positively charged nanoparticles have been shown to have shorter circulation half-life, but enhanced internalization due to their adsorptive interaction with the cell membrane. Interestingly, Yuan et al. demonstrated the enhanced tumoral retention of zwitterionic nanoparticles with switchable charge based on environmental stimulus (Yuan et al., 2012; Blanco et al., 2015). The i.v. injection of 0.5 nmol of NIR QDs in our models did not result in NIR QDs tumor detection via EPR effect under our experimental conditions (Supplementary Figure 1). This absence of EPR effect is unlikely to be due to QDs aggregation or agglomeration as these QDs have been shown to be stable and disperse in serum (Debayle et al., in review). One alternative to enhance the tumor targeting is to couple the nanoparticles to antibodies or peptides. For instance, RGD (Arg-Gly-Asp) is a triple-peptide motif with binding affinity to α_v_β_3_ integrin which is highly expressed in neovasculature and many tumor lines (Martelli et al., 2016). Optical and nuclear imaging probes coupled to RGD have been shown to target tumors and improve their visualization in mice (Li et al., 2006). Likewise, Trapiella-Alfonso et al. observed a higher tumor uptake of RGD-NIR QDs 15–30 min after probe injection, as well as, similarly to us, a lower long lasting fluorescence with non-functionalized NIR QDs (Trapiella-Alfonso et al., 2018). In addition, NIR QDs or iron oxides nanoparticles coupled to anti-HER2 showed high specificity in targeting subcutaneous ovarian and prostate xenografts (Gao et al., 2011). The signal localization and intensity hence depend on several physiological parameters including mainly the tumor vascularization and its environment. It would be interesting to investigate in more details what are the critical parameters enabling QDs tumor uptake with different types of functionalization as well as different tumor models providing different patterns of vascularization.

To summarize and conclude, we have shown the development of two different tumor models and FUEL ability to red shifting their emission. With further improvements, this optical method could offer an attractive alternative for detecting smaller and deeper tumors in preclinical models.

## Author Contributions

FS, SB, ISA, SLS, and RT designed the study. FS and SB performed the *in vitro* and *in vivo* experiments. TP and NL synthetized, characterized, and provided the quantum dots. FS, SB, AD, J-YT, ISA, SLS, and RT analyzed and interpreted the data. SLS and RT supervised the study. FS, SB, SLS, and RT wrote the manuscript. All authors read and approved the final manuscript.

### Conflict of Interest Statement

The authors declare that the research was conducted in the absence of any commercial or financial relationships that could be construed as a potential conflict of interest.
