# Supplementary material for: Imaging of Red-Shifted Light From Bioluminescent Tumors Using Fluorescence by Unbound Excitation From Luminescence
Source: Front Bioeng Biotechnol. 2019 Apr 5;7:73. doi: 10.3389/fbioe.2019.00073 (PMC6460942; doi:10.3389/fbioe.2019.00073)

**Supporting information legends**

**S1 Fig. Absence of QD EPR effect. A)** Fluorescence quantification in tumors and abdominal control regions. B) representative image of fluorescence 24hours post QDs injection showing tumors and control ROI.


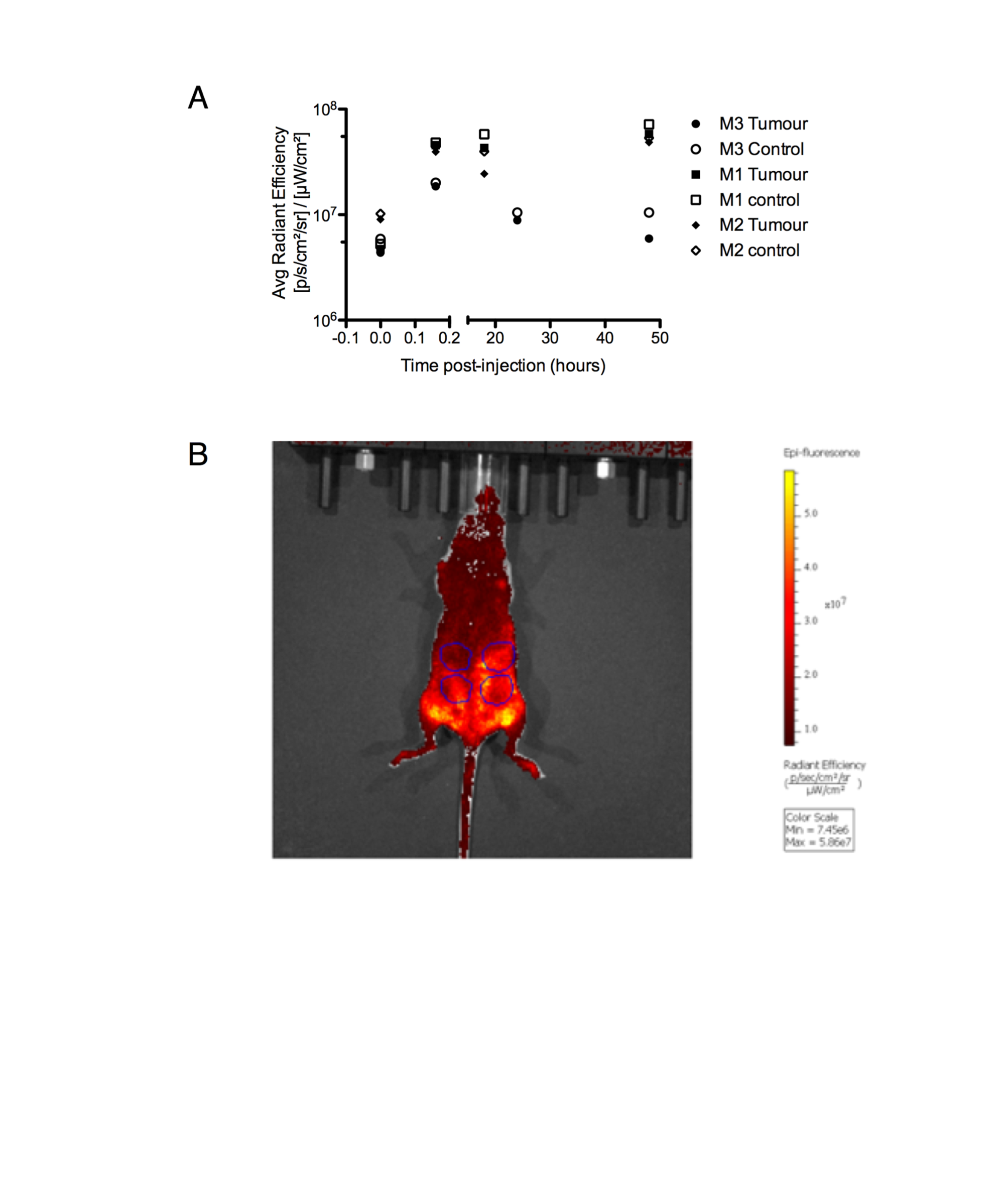

Supplement: Supplementary file 1 [file Table_1.DOCX]
